# Undernutrition and associated factors among school-age children in Wolaita Zone, South Ethiopia: a comparative cross-sectional study

**DOI:** 10.3389/fnut.2024.1400276

**Published:** 2024-09-18

**Authors:** Dereje Kutafo Meskele, Temesgen Lera Abiso, Tinsae Bekele Belete, Mengistu Meskele Koyira, Samson Kastro Dake

**Affiliations:** ^1^Maternal, Child and Nutrition Department, Kindo Didaye District Health Office, Halale, Ethiopia; ^2^School of Public Health, College of Health Sciences and Medicine, Wolaita Sodo University, Wolaita Sodo, Ethiopia; ^3^Department of Public Health, Tarch Campus, Wolaita Sodo University, Dawuro, Ethiopia

**Keywords:** under-nutrition, school-age children, school feeding program, Kindo Didaye district under-nutrition, Non-school feeding program

## Abstract

**Background:**

Nutritional deficiencies in school-age children are a public health concern, especially in resource-limited countries. A school feeding program involves the provision of food on-site or taken home to reduce hunger. It is implemented in several developing nations; however, little is known about the association of school feeding programs with the nutritional status of school-age children in the study area.

**Objectives:**

The study aimed to determine the magnitudes and associated factors of undernutrition among school-age children with school feeding programs (SFPs) and non-school feeding programs (N-SFPs) in Kindo Didaye woreda, South Ethiopia.

**Methods:**

A school-based comparative cross-sectional study was conducted in Kindo Didaye district from May to June 2023. A total of 612 participants were included in the study. The data were collected from each selected student's parents by using a structured interviewer-administered questionnaire. The weight and height of the children were measured, and a household dietary diversity assessment was conducted. The data were analyzed using SPSS version 25. A binary logistic regression analysis was carried out. A *p*-value of <0.05 and 95% confidence interval (CI) were used to establish a statistically significant association.

**Results:**

The magnitude of undernutrition among the school-age children was 38.9%: 43.3% in the children from the SFP schools and 34.5% in the children from the N-SFP schools. Stunting was 24.1% among the children in the schools with SFPs and 16% among the children in the N-SFP schools, whereas thinness was 33.8% among the children in the SFP schools and 25.6% among the children in the N-SFP schools. The children who were in the older age group [adjusted odds ratio (AOR) = 4.4, 95%CI; 2.22–8.85], consumed less than three meals per day at home (AOR = 6.03; 95%CI 3.9–9.3), and did not eat breakfast at all before going to school (AOR = 3.5; 95%CI 1.15–10.76) were more likely to become undernourished. The children whose fathers received secondary and above education (AOR = 0.52; 95% CI (0.27–0.971) had lower odds of becoming underweight.

**Conclusion:**

The magnitude of undernutrition was high in the current study. Existing interventions that work to improve the nutritional status of school-age children should be strengthened. Children should consume any type of food as breakfast at home before going to school regardless of the presence of school feeding programs and at least three times a day.

## Introduction

Undernutrition results from inadequate intake of energy, protein, and micronutrients. It can also be caused by poor absorption or quick loss of nutrients owing to disease or excessive energy use. It is characterized by low birth weight, stunting, wasting, underweight, and micronutrient deficiencies (1, 2). School age is characterized by dynamic physical growth, mental development, and a high vulnerability stage (3). For a child who is severely malnourished, attending school is less crucial than getting enough food to eat. Children are more likely to participate in school if they are guaranteed at least one healthy meal daily. Human development progresses quickly when nutritional needs increase and dietary habits are established. To prepare for the body's rapid growth during adolescence, it is also the ideal time to increase nutrient storage. Being well-nourished during school age is essential because poor nutrition during childhood can cause malnutrition, growth impairment, decreased work ability, and poor mental and social development (4, 5).

Nutritional deficiencies in school-age children are a public health concern, especially in resource-limited countries. Stunting and thinness, which have severe consequences for survival and health and the development of school-age children, most commonly affect children in low- and middle-income countries. Approximately 52.0% of school-age children in such countries are stunted (5, 6).

According to the Global Education Monitoring Report, more than a quarter of children under the age of 15 living in Sub-Saharan Africa (SSA) were thin. The global prevalence of malnutrition among school-age children (5–15 years old), as indicated by the prevalence of stunting, was approximately 28% (171 million children), with Eastern Africa experiencing a higher rate of 45 % (7, 8).

Malnutrition among school-age children in developing nations, especially in Africa, has been linked with morbidity, hygienic practices, dietary intake, and family socioeconomic status (9–11). It is a major public health problem (12, 13). It is evident that a significant percentage of school-age children experience undernutrition and that there was no nutrition intervention for addressing the problem among school-age children in Ethiopia until 1994 (9). Different strategies, programs, policies, and interventions were adopted at international and national levels to minimize the burdens of children's nutritional status (8).

School feeding is the provision of food either on-site or to take home. In middle- and low-income countries, school feeding programs (SFPs) have two different branches of aims. In the short term, it aims to alleviate hunger, exists as a social safety net for households with very low income, increases enrolment, promotes regular attendance to improve overall performance, and reduces temporary hunger in schools (2, 14). In the long term, it aims to improve the nutritional status, attendance, cognitive development, and retention of school children (15). School-feeding programs (SFPs) have been continuously gaining popularity in developing countries, mainly among those that are severely affected by childhood hunger and malnourishment. Currently, SFPs exist in 70 of the 108 low-income and middle-income countries, and most of them have been initiated and funded by the World Food Programme.

In Ethiopia, a World Food Programme-sponsored school feeding program was started in 1994, with an initial pilot project in the war-affected zones in the Tigray region. Presently, the Ethiopian SFP provides school meals for students in six regions of the country (Afar, Amhara, Oromia, Somali, Tigray, and the Southern Nations, Nationalities, and Peoples' Region). In the country, the target areas for implementing SFPs were woreda (the third-level administrative divisions in the country), with chronic food insecurity, lower school enrolment, and higher gender disparity (14, 16). However, there is no sufficient evidence on the effect of school feeding programs on the nutritional status of school children and its associated factors, especially in Ethiopia, particularly in the study area. Therefore, this study aimed to assess the magnitudes and associated factors of undernutrition among school-age children enrolled in schools with school feeding programs and non-school feeding programs (N-SFP) in Kindo Didaye district, South Ethiopia.

## Methods

### Study area and period

Kindo Didaye district is one of the 16 districts in Wolaita Zone, South Ethiopia. The district is located 86 km from Wolaita Sodo city, the zonal administration center, and 475 km from Addis Ababa, the capital city of Ethiopia. The district has 19 kebeles (smallest administrative unit), where 17 are rural and two are urban kebeles, with an estimated total population of 96,120. The district has a total area of 38,045.7 hectares (17). The inhabitants of the district are primarily dependent on agriculture, and the typical meals in the district include kocho, which is manufactured from fake bananas (*Ensete ventricosum*), kita (primarily maize), cassavas, roots, and cereals. Teff, wheat, cassava, barley, and maize are popular crops typically grown between May and July and harvested between September and November (17). The school feeding program has been placed in 8 of the 28 primary schools in the woreda since 2020/21Gc. The total number of primary school children is 20,074; among those, 10,541 are male students and 9,533 are female students (17). The study was conducted from 1^st^ May to 20^th^ June 2023.

### Study design

A school-based comparative cross-sectional study design was employed.

### Sample population

All school-aged children in all primary schools of the woreda were the source population, all school-age children in the selected primary schools both with SFPs and N-SFPs of the woreda were the study population, and the selected school-age students (5–15 years) in the selected primary schools both with SFPs and with N-SFPs were the sample population.

### Inclusion criteria and exclusion criteria

#### Inclusion criteria

Students aged between 5 and 15 years who attended school during the data collection in the schools with and without school feeding programs were included in this study.

#### Exclusion criteria

Severely ill children who could not be involved in the data collection due to an illness were excluded during the data collection period.

### Sample size determination and sampling technique

The sample size was calculated using G-power software. The required sample size of the study was calculated after comparing the prevalence of stunting, thinness, and the proportion of strongly associated factors such as sex, monthly income, and having a farmland; the proportion that resulted in the maximum sample size was selected. The proportion of stunting was 58.5% among the students who did not take meals at school and 48.3% among the students who took meals at school from a study conducted in the Meket district (18). In addition, the sample size was calculated by considering the following assumptions: 95% confidence interval (CI) and 80% power, a margin of error of 5%, a population allocation ratio of 1:2, a design effect of 1.5, and a 10% non-response rate. The final sample size calculated was 616.

### Sampling technique

**Figure 1:**
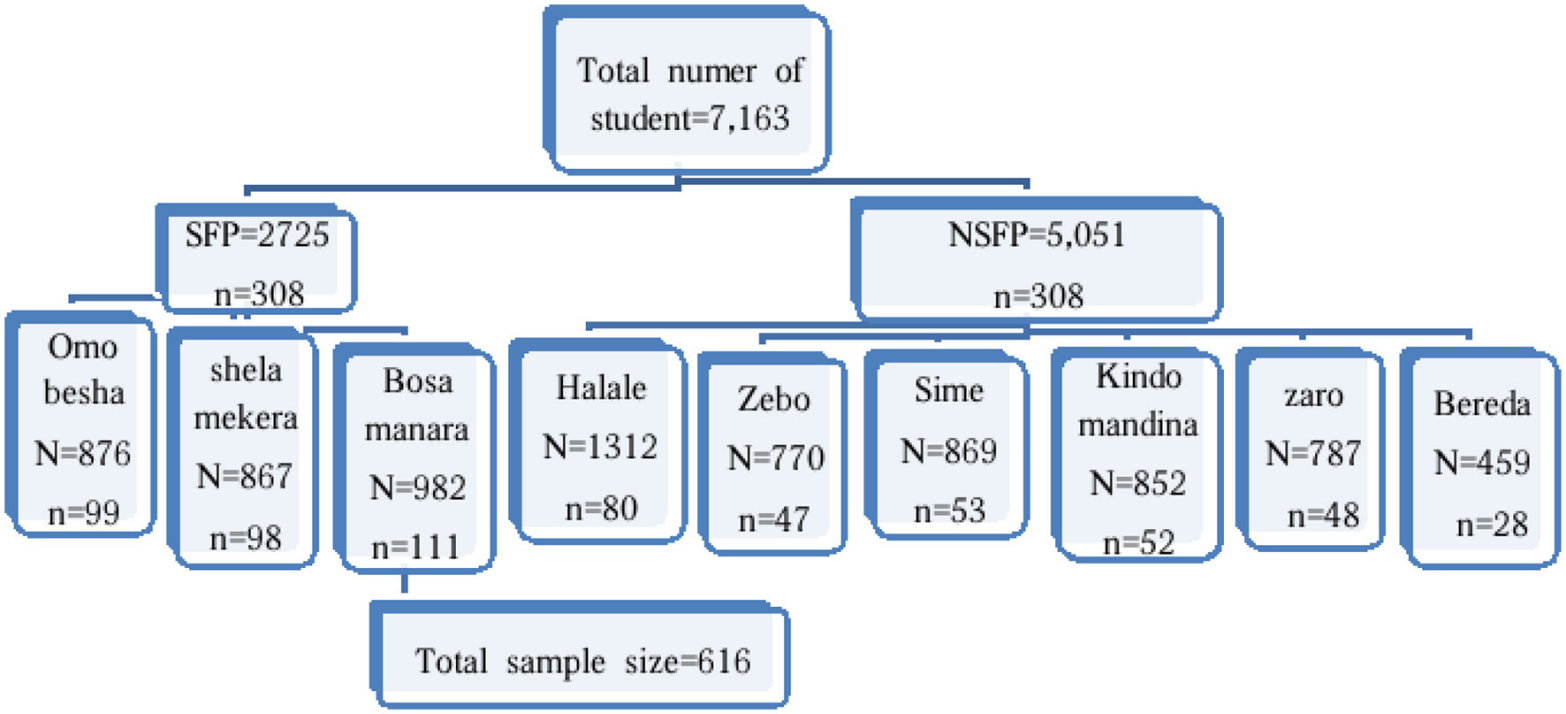
Schematic representation of the sampling procedure both in the SFP and N-SFP schools in Kindo Didaye woreda in 2023.

In Kindo Didaye district, there are 28 primary schools. First, the schools in the woreda are stratified as those implementing SFPs (eight in number) and those not implementing SFPs (20 in number). Then, three from the SFP schools and six from the N-SFP schools, with a ratio of 1:2, were selected using a simple random sampling method. The calculated sample size was proportionally allocated to the selected schools based on the proportion to the size of the students from each school. The students who fulfilled the inclusion criteria were screened by the school administrators before the selection for the study. Then, the screened school-age students were coded with their grades and sections. Finally, using the student list as a sampling frame, the students were selected by the simple random sampling technique from each selected school (Figure 1).

### Study variables

The outcome variable was undernutrition, and the exposure variables included socio-demographic and economic variables, health status, individual dietary intake, and sanitation and hygiene conditions.

### Data collection tools and measurements

The questionnaire was adapted after reviewing related previous literature (11, 14, 18–22). The data were collected using a structured interviewer-administered questionnaire via Kobo Collect software 2023.1.2 a. A part of the dietary diversity score (DDS) questionnaire was adapted from the Food and Agriculture Organization of the United Nations and other related literature (11, 23, 24). The children's dietary diversity was assessed according to their mothers' or caregivers' responses regarding whether the children had eaten different food groups from yesterday's sunrise to today's sunrise (24-h recall method) before the survey date. A child with a DDS of four and above was classified as having good dietary diversity; other scores were classified as poor. Consuming food groups within the range of 1–3 suggests lowest dietary diversity, consuming food groups within the range of 4–5 suggests medium dietary diversity, and consuming food groups within the range of 5–7 suggests high dietary diversity (25).

The weight and height of the students were measured using a weighing scale and stadiometer, respectively. A portable digital flat Seca scale (Seca electronic scale, 770 Hamburg) and a Seca body meter (Seca 274 body meter) were used to measure the weight and height, respectively. During the weight measurement, the weighing scales were calibrated each day before the actual data collection using a known weight material. The weight was measured to the nearest 0.1 kg using a digital scale. The scale was adjusted before weighing every student by setting it to zero. The students were lightly dressed while having the weight taken. The height was measured to the nearest 0.1 cm. While measuring the height, each student stood in a normal anatomical position without the shoes, heels, buttocks, shoulder, and back of the head touching the measuring board. Then, the headpiece of the measuring board was moved to touch the top of the head. For both weight and height, two readings were recorded. The z-score values for body mass index (BMI)-for-age and height-for-age were calculated using the WHO AnthroPlus software. The calculated z-scores of the BMI-for-age and height-for-age were used to classify thinness and stunting using the new WHO 2007 reference values.

### Operational definition

#### Undernutrition

This composite outcome or dependent variable was generated by summing up the variables stunting and thinness. It is defined as having stunting, thinness, or both.

#### Stunting (Chronic malnutrition)

It refers to height-for-age z-score (HAZ) < −2 SD of the median value of the WHO AnthroPlus, 2007, international growth reference (26).

#### Thinness (Acute malnutrition)

It refers to BMI-for-age z-score (BAZ) < −2 SD of the median value of the WHO AnthroPlus, 2007, international growth reference (26).

#### Minimum dietary diversity score

It included the proportion of the school-aged children who received foods from four or more food groups of the seven food groups over 24 h before the survey. Consuming food groups within the range of 1–3 suggests lowest dietary diversity, consuming food groups within the range of 4–5 suggests medium dietary diversity, and consuming food groups within the range of 5–7 suggests high dietary diversity (25).

#### School age

The school age comprised 5–15 years (27).

#### Absenteeism rate

This was determined as the number of days a child was absent from school in the last 2 weeks of the survey.

### Data quality management

The English version of the structured questionnaire was translated into the local language, Wolaitta Doonaa, and back to English by a language expert for ensuring the consistency of the questions. The pretest (5%) was conducted at Bele Awasa and Dinsa primary schools in Kindo Koysha district. To ensure the accuracy and precision of the measurements, the data collectors were trained for 2 days on how to administer the questionnaire, use Kobo Collector version 2023.1.2, and conduct anthropometry in a standardized manner. The weighing scale calibration with a standard weight was performed daily during the data collection. Each day after the data collection, the questionnaires were reviewed to ensure the completeness of the data.

### Data analysis

The data were exported to Excel from the KoboCollect tool and then to SPSS version 25 software for analysis. Descriptive statistics such as frequency, proportion, measures of central tendency, and measures of dispersion were computed. A logistic regression model was carried out for both bivariable and multivariable analysis for identifying factors associated with undernutrition. A *p*-value ≤ 0.25 was taken as the cut-off point for selecting variables for the final model. An adjusted odds ratio (AOR) with its respective 95% CI was used to establish a statistically significant association at a *p-*value < 0.05. After checking for multicollinearity by using a variance inflation factor, a variance inflation factor of < 10 was considered the minimum threshold for collinearity. The model fitness was checked using the Hosmer and Lemeshow model fitness.

### Ethical considerations

Ethical clearance was obtained from the Institutional Review Committee of Wolaita Sodo University, with an ethical review number of CHSM/ERC/01/15, and a permission letter was obtained from the Kindo Didaye district education office for the data collection. From the participants involved, verbal informed consent and assent were taken; the right to not participate was respected, and the obtained information was confidential.

## Results

### Participants' socio-demographic and socioeconomic characteristics

A total of 612 school children were involved in the study, which made the response rate 99%. Of the 305 students from the schools with SFPs, 188 (53.1%) were male and 117 (46.9%) were female. Of 307 participants from the schools with N-SFPs, 163 (61.6%) were male and 144 (38.4%) were female. The median age of the school children was 12, with an interquartile range of 3 years. The majority of the school children, both male and female, were in the 10–15 age category, 248 (80.8%) and 280 (91.8%), respectively (Table 1).

**Table 1:** Socio-demographic and economic characteristics of the participants in the schools with SFPs and non-SFPs in Kindo Didaye district, 2023.

**Variable (*n =* 612)**		**N-SFP**		**SFP**	
	**Options**	**Frequency**	**Percent%**	**Frequency**	**Percent%**
Sex of the children	Male	163	53.1	188	61.6
	Female	144	46.9	117	38.4
Age of the children	5–9	59	19.2	25	8.2
	10–15	248	80.8	280	91.8
Grade of the children	1–4	154	50.2	168	55.1
	5–8	153	49.8	137	44.9
Household family size	≤ 3	26	8.5	14	4.6
	>3	281	92.5	291	95.4
Child's caregiver	Mother	198	64.5	229	75.1
	Others^*^	109	35.5	76	24.9
Father's educational status	Did not attend formal education	117	38.1	180	59.0
	Primary	80	26.1	63	20.7
	Secondary	46	15.0	29	9.5
	Above secondary	57	18.6	16	5.2
Mother's educational status	Did not attend formal education	161	53.7	228	76.3
	Primary	72	24.0	36	12.0
	Secondary	33	11.0	22	7.4
	Above secondary	34	11.3	13	4.3
Father's Occupation	Farmer	156	51.8	230	77.2
	Others^*^	145	48.2	68	22.8
Mother's Occupation	Housewife	154	51.2	219	73
	Others^*^	147	48.8	81	27
Monthly income of the household (Ethiopian Birr)	< 1,000	137	44.6	216	70.8
	1,001–2,500	108	35.2	57	18.7
	≥2,501	62	20.2	32	10.5
Marital status of the caregiver	Currently married	283	92.2	280	91.8
	Currently not married^*^	24	7.8	25	9.2
Mother's age	20–34	131	42.7	121	39.7
	35–44	121	39.4	89	30.1
	≥45 years	51	16.8	86	29.1
Birth order of the child	≤ 2	149	48.5	115	37.7
	≥3	158	51.5	190	62.3
	No	57	18.6	29	9.5
	≥10	15	6	13	4.3
Having farming land and cultivated products	Yes	227	73.9	305	100
	No	80	26.1	0	0
Having domestic animals	Yes	250	81.4	276	90.5
	No	57	18.6	29	9.5

^*^Other caregivers were, father, aunt, grandmother, grandfather, sister, and brother. ^*^Other occupations of the fathers and mothers were student, self-employed, government-employed, housewife, and daily laborer. Other marital status ^*^Not married, divorced, widowed, and single.

### Dietary history of the participants

All students enrolled in the schools with SFPs ate once in school, and each student got 150 g of a meal prepared from wheat, nifro, kimchi, rice, pasta, and macaroni once a day from Monday to Friday. The majority of the school children on the school feeding program were found to eat < 2 times at home in a day, while those in the schools without a school feeding program were found to eat comparatively less. The majority, 188 (61.2), of the children from the schools without a school feeding program were found to take their breakfast at home every morning before going to school, while nearly a third, 111 (36.3%), of the children from the schools with a school feeding program were found to go to school without eating breakfast (Table 2).

**Table 2:** Feeding characteristics of the children from both SFP schools and N-SFP schools in Kindo Didaye district, 2023.

**Variables (*n =* 612)**	**Options**	**School types**			
		**N-SFP**		**SFP**	
		**Frequency**	**%**	**Frequency**	**%**
The child eats breakfast before going to school	Never	9	2.9	19	6.2
	Yes, 3–4 days per week	110	35.8	175	57.4
	Yes, every day	188	61.2	111	36.4
The number of meals the child ate at home in the past 24 h before the survey	≤ 2 Meal	89	29	102	33.4
	>3 Meal	218	71	203	66.6

SFP, School feeding program; N-SFP, Non-school feeding program.

**Figure 2:**
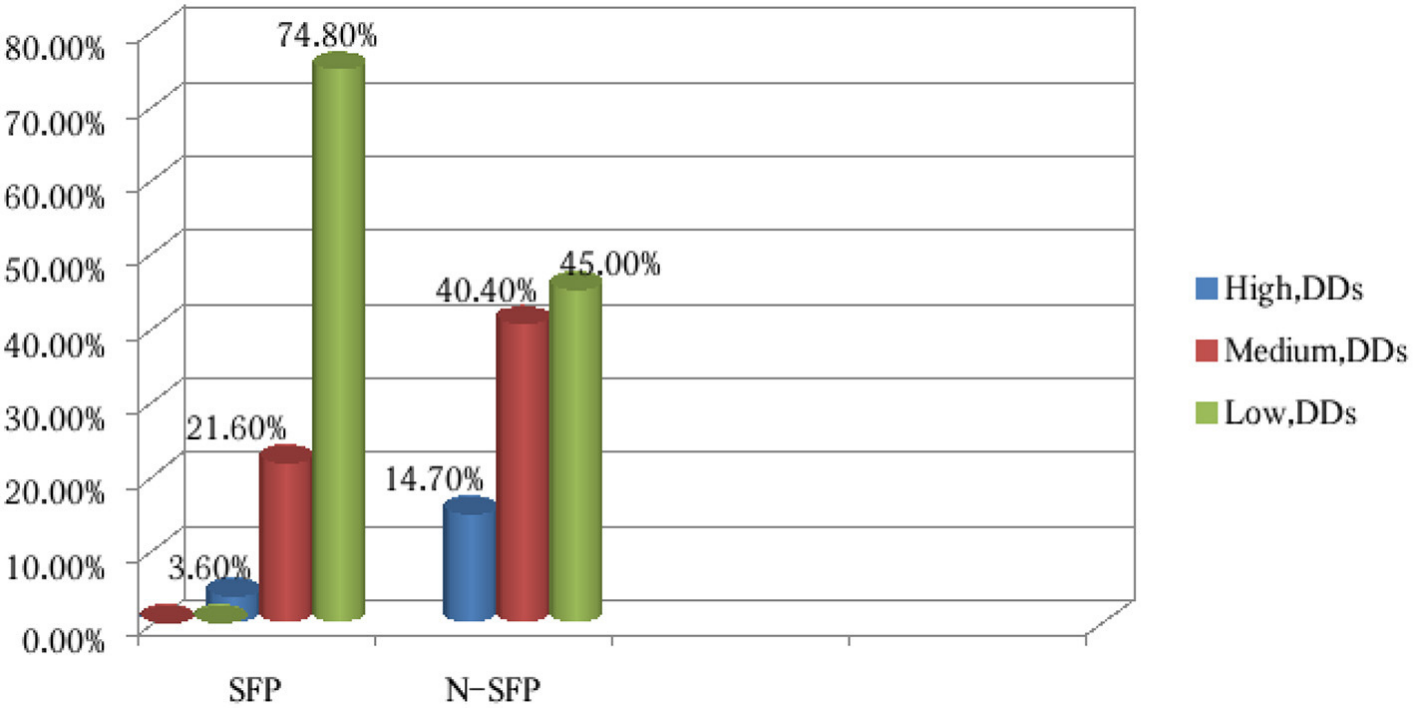
Dietary assessments of the last 24 h before the survey of the children from the SFP schools and N-SFP schools in Kindo Didaye woreda, 2023 (*n* = 612).

According to the 24-h dietary recall, the number of food groups eaten within 24 h before the survey by the majority of the students from the schools with a school feeding program was < 3, approximately 228 (74.8%), while nearly half of the children from the non-school feeding program schools ate more than three food groups within 24 h (Figure 2). The dietary intake of the school children mainly consisted of cereals, pulses, legumes, nuts, roots, tubers, other fruits, and vegetables compared to animal source foods and green leafy vegetables. In the study, the students from both the schools with SFPs and the non-school feeding program schools were less likely to consume food groups such as meats, eggs, and vitamin A-rich fruits and vegetables.

### Participants' incidence of ill health

The number of children who had fallen sick within 2 weeks before the data collection, as reported by the caregivers, was 89 (29.2%) from the schools with school feeding programs and 70 (28.2 %) from the non-school feeding program schools. Fifty-one (16.7%) children from the schools with SFPs and 28 (9.1%) children from the non-SFP schools had diarrhea 2 weeks before the data collection. Following episodes of malaria 2 weeks preceding the survey, the parents of 46 (15.1%) children in the schools with a school feeding program and of 34 (11.1 %) children in the N-SFP schools reported that their children had fever (Table 3). Malaria and other infections present symptoms such as fever. Malaria being endemic in these communities could be the reason why both groups mentioned fever as the primary ailment in their children.

**Table 3:** The incidence of ill health among the children in the last 2 weeks before the survey in both SFP and N-SFP schools in Kindo Didaye district, 2023.

**Variable (*n =* 612)**	**N-SFP**		**SFP**	
	**Frequency**	**Percent (%)**	**Frequency**	**Percent (%)**
**The child had fallen sick in the last 2 weeks**				
Yes	70	28.2	89	29.2
No	237	77.8	216	70.8
**The child had a history of diarrhea in the last 2 weeks**				
Yes	28	9.1	51	16.7
No	279	80.9	254	83.3
**The child had a history of malaria in the last 2 weeks**				
Yes	34	11.1	46	15.4
No	273	88.9	259	84.6
**The child had a history of cough in the last 2 weeks**				
Yes	16	5.2	15	4.9
No	291	94.8	290	95.1
**The child was dewormed for intestinal parasites in the last 6 months**				
Yes	85	27.7	58	19
No	222	72.3	247	81

SFP, School feeding program; N-SFP, Non-school feeding program.

### Participants' sanitation and hygiene

All the schools had latrines, but none of them had hand-washing facilities. The majority of the households of the students from both groups had a latrine (99% and 96.1%), and the type of latrine used in the majority of the households of the students from both groups was a pit latrine without a slab, 286 (93.7%), and only two households from the SFP group and one household from the N-SFP group had a ventilated pit latrine. Twelve households from the SFP group and three households from the N-SFP group had no latrines and used open fields for defecation. The primary source of drinking water for 204 (66.9%) households of the children from the SFP group and for 226 (73.4%) households of the children from the N-SFP group was protected sources such as tap water. Protected springs as a source of drinking water were found in 75 (24.6%) of the households of the children from the SFP group and 70 (22.8%) of the households of the children from the N-SFP group. However, 8.6% and 3.6% of the households of the students from the SFP group and the N-SFP group, respectively, sourced their drinking water from unprotected sources, such as unprotected springs, ponds, and surface water. Approximately 58.9% and 61.6% of the students from the N-SFP group and the SFP group, respectively, were found to wash their hands before eating food. However, more than half of the children in both groups did not always wash their hands with soap after using the toilet (Table 4).

**Table 4:** Sanitation and hygiene condition of the participants in both SFP and N-SFP groups in Kindo Didaye district, 2023.

**Variable (*n =* 612)**	**N-SFP**		**SFP**	
	**Frequency**	**Percent (%)**	**Frequency**	**Percent (%)**
**Schools supply safe water for students**				
Yes	82	26.7	185	60.7
No	225	73.3	128	39.3
**Children wash their hands with soap before eating food**				
Always	179	58.3	188	61.6
Sometimes	128	41.7	117	38.4
**The household toilet has water for hand-washing with soap**				
Yes	53	17.3	48	16.4
No	254	82.7	245	83.6
**Type of household latrine**				
Pit latrine with a slab	17	5.5	5	1.7
Pit latrine with no slab/open pit	289	94.1	286	97.6
**Children wash their hands with soap after using the toilet**				
Always	83	27	54	17.7
Sometimes	182	59.3	187	61.3
Never at all	42	13.7	64	21
**The household's main source of drinking water**				
Tap water	226	73.6	204	66.9
Spring (protected)	70	22.8	75	24.6
Spring (unprotected)	11	3.6	26	8.5
**Time to rich to collect drinking water source from children's houses in minutes**				
≤ 15	165	53.7	141	46.2
16–30	104	33.9	133	43.6
≥30	38	12.4	31	10.2

SFP, School feeding program; N-SFP, Non-school feeding program.

### Nutritional status of the school-age children

**Figure 3:**
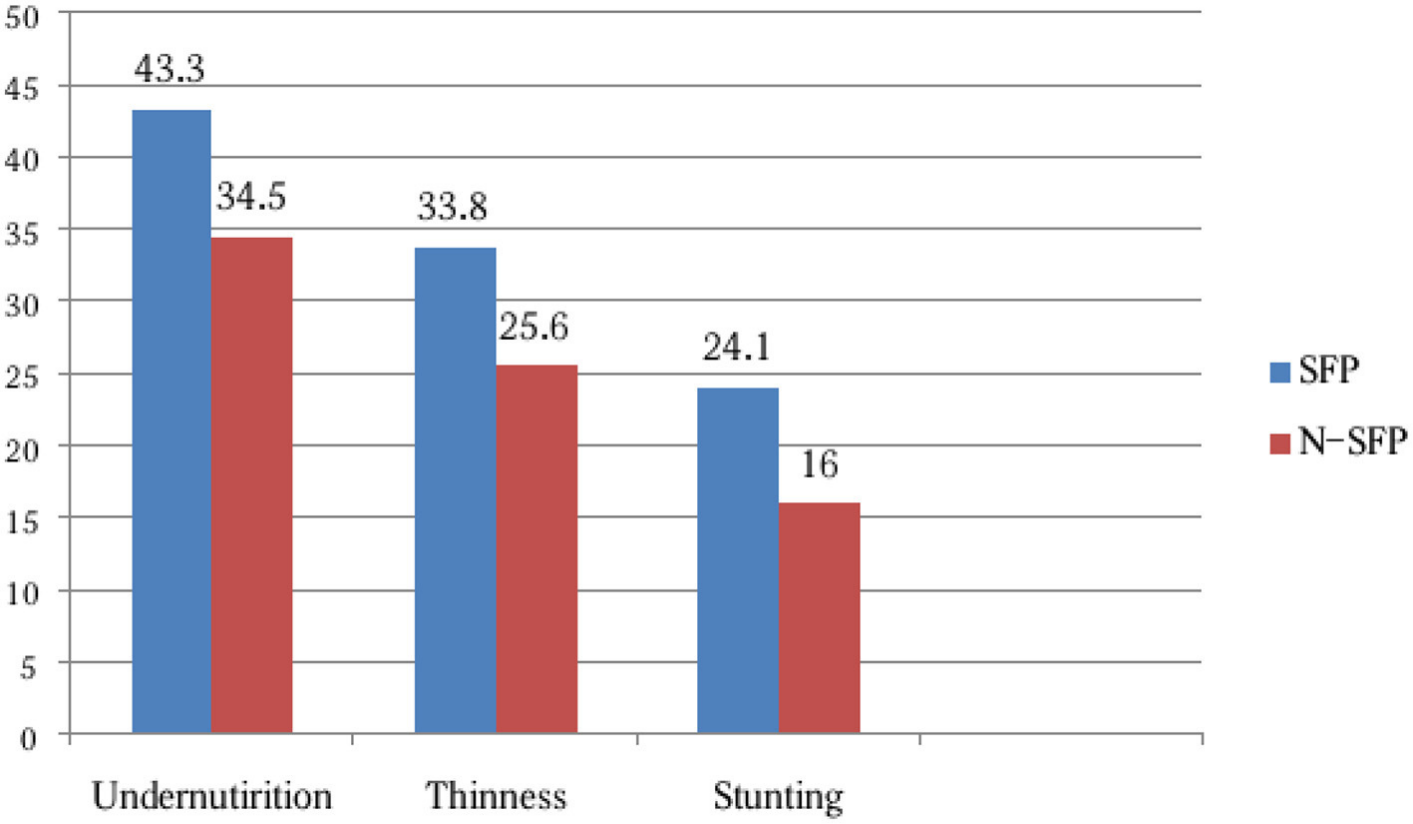
Nutritional status of the school-age children in both SFP and N-SFP groups in Kindo Didaye district, 2023 (*n* = 612).

The overall prevalence of undernutrition was observed in 238 (38.9%) children in total, 132 (43.3%) among the children in the schools with an SFP and 106 (34.5%) among the children in N-SFP schools. The magnitude of thinness was observed in 33.8% of the children in the schools with an SFP and in 25.6% of the children in the N-SFP schools. In addition, the prevalence of stunting was observed in 24.1% of the children in the schools with an SFP and in 16.0% of the children in the N-SFP schools (Figure 3).

### Factors associated with undernutrition in both SFP and N-SFP schools

In the multivariable logistic regression model, the age of the child, the father's educational status, eating breakfast before going to school, and the frequency of meals were significantly associated with undernutrition. The children in the older age group (10–15 years) were 4.4 (AOR = 4.4, 95% CI: 2.22, 8.85) times more likely to be undernourished than the children in the younger age group. In addition, the odds of undernutrition among the children whose fathers received secondary education and above was 48% (AOR = 0.52, 95% CI: 0.27, 0.97), which was lower compared to the children whose fathers did not receive formal education. In addition, the children who never ate breakfast before going to school were 3.5 (AOR = 3.5, 95% CI: 1.15, 10.76) times more likely to be undernourished than those who always ate breakfast before school. The odds of undernutrition were six (AOR = 6.0, 95% CI: 3.91, 9.32) times higher among the children who consumed two or fewer meals a day than the children who consumed more than two meals a day (Table 5).

**Table 5:** Multivariable logistic regression analysis of the factors associated with undernutrition of the children in both SFP and N-SFP schools in Kindo Didaye district, 2023.

**Variables (*****n*** **=** **612)**		**Undernutrition**		**COR**	**AOR**
		**Yes**	**No**	**(95% CI)**	**(95% CI)**
Sex	Male	169	222	1	1
	Female	69	152	0.2(0.51, 0.92)	1.7(0.42, 2.40)
Age	5–9	11	73	1	1
	10–15	227	301	5.0(2.59, 9.65)	4.4(2.22, 8.85)^**^
Family size	≤ 3	11	29	1	1
	>3	227	345	1.7(1.84, 3.52)	2.4(0.84, 7.41)
Father's educational status	No education	129	170	1	1
	Primary	69	147	0.6(0.42, 0.89)	0.6(0.28, 1.18)
	Secondary and above	31	44	0.9(0.55, 1.51)	0.5(0.27, 0.97)^*^
Mother's educational status	No formal education	177	230	1	1
	Primary	75	75	1.6(0.77, 3.3)	1.6 (0.71–4.02)
	Secondary and above	12	33	5.3(1.8, 16.22)	1.2(0.46, 2.93)
Father's occupation	Farmer	157	213	1	1
	Other	70	143	0.8(0.52, 1.22)	0.8(0.51, 1.42)
Mother's Occupation	Housewife	150	204	1	1
	Other	80	148	0.7(0.52, 1.03)	0.9(0.56, 1.43)
Having domestic animals	Yes	211	315	1	1
	No	27	59	0.7(0.11, 0.42)	1.4(0.73, 2.88)
The child eats breakfast before school	Always	102	197	1	1
	Sometimes	118	167	1.3(0.97, 1.93)	1.1(0.69, 1.66)
	Never	18	10	3.4(1.54, 7.81)	3.5(1.15,10.76)^*^
Frequency of meals	≤ 2 times	86	105	1.4(1.02, 2.05)	6.0(3.91, 9.32)^**^
	>2 times	152	269	1	1
The child is a part of an SFP	Yes	132	173	0.7(0.49, 0.95)	1.0(0.66,1.62)
	No	106	201	1	1
Have a farmland and cultivated products	Yes	207	303	1.7(0.40, 0.83)	1.2(0.38, 4.06)
	No	31	71	1	1
The child had malaria in the last 2 weeks	Yes	37	43	1.9(1.01, 3.72)	0.7(0.37, 1.19)
	No	24	54	1	1
Dietary diversity	Low	184	235	1	1
	Medium	42	105	0.5(0.33, 0.75)	1.4(0.73, 2.83)
	High	12	36	0.4(0.21, 0.83)	1.0(0.66, 1.61)

COR, crude odds ratio; AOR, adjusted odds ratio; ^*^ *p*-value < 0.05, ^**^p-value < 0.001, CI, confidence interval. Above secondary level: certificate and diploma, degree, and above. Other occupations: student, self-employed, government-employed, housewife, and daily labor.

## Discussion

This study attempted to determine the prevalence of undernutrition and identify its associated factors. The results of the current study showed that the overall prevalence of undernutrition was 38.9%, 43.3% and 34.5% among the children in the SFP group and the N-SFP group, respectively. The study findings also revealed that the children from the older age group (10–15 years), the children whose fathers received secondary education and above, the children who never ate breakfast before school, and the children who consumed two or fewer meals a day were statistically significantly associated with undernutrition.

In this study, the overall prevalence of undernutrition was 38.9%. Undernutrition was higher (43.3%) among the children in the SFP schools as compared to the children in the N-SFP schools (34.5%). The magnitude was higher than the findings reported by studies conducted in Addis Ababa (31%), Durbete town (32.1%), and Ethiopia (12, 28). This finding was lower than the prevalence reported in Northwest Ethiopia, which was 41.6% and 71.98%, respectively (29, 30). The magnitude of thinness in the current study was 33.8% and 25.6% in the schools with SFP and in the N-SFP schools, respectively, whereas stunting was 24.1% and 16% in the schools with SFP and in the N-SFP schools, respectively. This result was higher than that of a study conducted in Southern Ethiopia, which revealed that the prevalence of thinness was 14.3% and 19.5% and that of stunting was 21.1% and 20.4% in SFP and N-SFP schools, respectively (31). The prevalence of stunting and thinness in Ethiopia's Dubti district in the Afar region was also lower than the results of the current study. In the abovementioned study, the prevalence of stunting in the SFP and N-SFP schools was 13.7% and 21.6%, respectively, and the prevalence of thinness was 4.9% in SFP schools and 13.9% in N-SFP schools (32). Similarly, a study in Kenya found that the prevalence of thinness was 12% in SFP schools and 11% in N-SFP schools, whereas the prevalence of stunting was 12% in SFP schools and 22% in N-SFP schools (9). In addition, the prevalence of thinness in this study was higher than that in the study demonstrated by Mohammed et al., which was 23.2% and 22.71% in SFP and N-SFP schools, respectively. However, the prevalence of stunting was lower than that in the same study, 31.5% and 26.3%, among the children in the SFP and N-SFP schools, respectively (11). These differences may be due to differences in the sample size, study setting, and study purposes.

In this study, it was found that there was no statistically significant association between school feeding programs and undernutrition. This finding was in line with other studies conducted in Southern Ethiopia (31) and Ghana (33), which showed that school feeding programs had no association with the nutritional status of children. Contrary to this, a study in Kenya (9) showed that school feeding programs had a negative association with the nutritional status of children. However, the result contrasted with studies conducted in different parts of Ethiopia (14, 18, 20, 32, 34), Kenya (35), Jamaica (36), and Ghana (21). The possible explanation for this discrepancy could be variations in the duration of intervention, availability of the required resources in schools, and the quality and quantity of foods served to children. Reports showed that in developing countries, school feeding programs were facing different challenges related to the quality and quantity of meals given to children (15, 37–39).

Furthermore, evidence from studies showed that SFPs have led to children being fed less at home. This is because some parents use the SFP as a replacement for feeding at home, even though it is meant to complement the child's diet along with home feeding (40). In addition, school feeding programs are implemented in selected schools located in areas where there is food insecurity (40).

The current study revealed that being in the older age group was positively associated with undernutrition. The odds of undernutrition were 4.4 times higher among the children aged between 10 and 15 years compared to the children aged 5 to 9 years. A similar finding was reported by a study conducted in different parts of Ethiopia (11, 16, 29, 34, 41–43). This might be due to experiencing a prolonged chronic food shortage. The consequence of undernutrition is a chronic nutritional problem, which develops over a relatively long period and is difficult to reverse once developed.

According to this study, the odds of undernutrition among the school children whose fathers received secondary education were 48% lower compared to the children whose fathers did not receive formal education. This finding was also similar to the findings from other studies conducted in South Ethiopia (42), Assam (44), Nepal (45), and India (6). This might be because literate parents adopt many improved behaviors related to maternal and child healthcare, feeding, and eating practices, which ultimately affect the nutritional status of children.

The odds of undernutrition among the children who never ate breakfast before school were 3.5 times those of undernutrition among the children who ate breakfast regularly before school. This was similar to previous studies in South Gondar Zone, Ethiopia (22), Kenya (3), and Nigeria (46). This could be because eating breakfast is crucial for getting enough nutrients and energy to prevent acute and chronic malnutrition in school-age children.

The frequency of meals that children take at home is another factor independently associated with undernutrition. The odds of undernutrition were six times higher among the children who ate < 3 meals a day compared to those who ate three or more meals a day. Previous studies in Meket Wereda, Ethiopia (18) in East Demibra district, Northwest Ethiopia (47), Nairobi, Kenya (3), and in Aladinma Owerri, Nigeria (46) showed similar results. This might be because those who eat < 3 times a day cannot meet the nutrient requirements.

This study used cross-sectional data, and the estimates might have been better represented if longitudinal follow-up data were used. Moreover, the school feeding intervention was not randomized; rather, schools were selected for the intervention based on the food insecurity and socioeconomic status of the area. Therefore, it is difficult to conclude that the school-feeding programs contributed to the higher undernutrition level. A certain level of recall bias is expected regarding the age and dietary intake. Interviewers who were aware of cultural issues collected the data to reduce recall bias.

## Conclusion

This study showed that the overall magnitude of undernutrition in the study area is high. Existing interventions that work to improve the nutritional status of school-age children should be strengthened. Children should consume any type of food as breakfast at home before going to school regardless of the presence of a school feeding program, and children should at least consume meals three times a day. We also recommend conducting longitudinal studies to explore further the effect of school feeding programs on the nutritional status of school-age children.

## Data Availability

The original contributions presented in the study are included in the article/supplementary material, further inquiries can be directed to the corresponding author.
